# Photoperiodic Regulation of Cerebral Blood Flow in White-Footed Mice (*Peromyscus leucopus*)

**DOI:** 10.1523/ENEURO.0058-16.2016

**Published:** 2016-07-27

**Authors:** Jeremy C. Borniger, Seth Teplitsky, Surya Gnyawali, Randy J. Nelson, Cameron Rink

**Affiliations:** 1 The Behavioral Neuroendocrinology Group, Wexner Medical Center, The Ohio State University, Columbus, Ohio 43210; 2 Department of Neuroscience, Wexner Medical Center, The Ohio State University, Columbus, Ohio 43210; 3 Neuroscience Research Institute, Wexner Medical Center, The Ohio State University, Columbus, OH 43210; 4 Department of Surgery, Wexner Medical Center, The Ohio State University, Columbus, Ohio 43210

**Keywords:** brain blood flow, hippocampus, *peromyscus*, photoperiod

## Abstract

Individuals living outside the tropics need to adjust their behavioral and physiological repertoires throughout the year to adapt to the changing seasons. White-footed mice (*Peromyscus leucopus*) reduce hippocampal volumes, hippocampal-dependent memory function, long-term potentiation, and alter neurogenesis in response to short (winter-like) day lengths (photoperiods). During winter, these mice putatively shunt energy away from the brain to maximize peripheral thermogenesis, immune function, and survival. We hypothesized that these changes in brain function are accompanied by alterations in brain vasculature. We maintained white-footed mice in short (8 h light/16 h dark) or long (16 h light/8 h dark) photoperiods for 8–9 weeks. Mice were then perfused with fluorescein isothiocyanate (FITC)-conjugated tomato (*Lycopersicon esculentum*) lectin to visualize the perfused cerebrovasculature. Short-day mice reduced hippocampal and cortical capillary density (FITC^+^ area); vessels isolated from short day-exposed mice expressed higher mRNA levels of the gelatinase matrix metalloproteinase 2 (MMP2). Additionally, short-day mice reduced cerebral blood flow ∼15% compared with their long-day counterparts, as assessed by laser speckle flowmetry. Immunohistochemistry revealed higher levels of MMP2 in the hippocampus of mice maintained in short days compared with long days, potentially contributing to the observed vascular remodeling. These data demonstrate that a discrete environmental signal (i.e., day length) can substantially alter cerebral blood flow in adult mammals.

## Significance Statement

Individuals living in nontropical climates show seasonal changes in physiology and behavior primarily controlled by day length (photoperiod). Specifically, white-footed mice (*Peromyscus leucopus*) display reduced hippocampal function, neurogenesis, and cognitive capacity in response to short days. It is unknown, however, whether these changes are preceded or accompanied by alterations in cerebral blood flow. This study provides evidence that short days elicit a reduction in hippocampal and cortical blood flow, and that this reduction is accompanied by increased mRNA and protein expression of the gelatinase matrix metalloproteinase 2, a primary component in vascular remodeling. These results have broad implications for our understanding of postnatal brain plasticity, environmental modulation of behavior, and seasonal changes in brain function.

## Introduction

Adult mammalian brains have some level of plasticity; however, environmental contributors to brain plasticity remain poorly understood. Nontropical rodents display seasonal variation in many aspects of their physiology and behavior (for review, see Follett, 2015), providing attractive models in which to study brain plasticity, development, and natural variation (Vrana et al., 2014; Bedford and Hoekstra, 2015). White-footed mice (*Peromyscus leucopus*) are among the most well studied photoperiodic small rodents (Lynch, 1973; Johnston and Zucker, 1980; Demas et al., 1996; Walton et al., 2014; Sharp et al., 2015;). Many seasonal changes in their physiology and behavior are driven by predictable changes in photoperiod (day length) across the year. For instance, in response to autumnal short day (SD) lengths, many white-footed mice regress their reproductive systems and reputedly shift energy toward survival, although there is substantial variation among populations living at different latitudes (Dark et al., 1983; Pyter et al., 2005a). Pineal melatonin plays an important role in this process; most short photoperiod-dependent phenotypic changes can be recapitulated under long-photoperiod conditions via the administration of exogenous melatonin (Bartness et al., 1993; Goldman, 2001; Hiebert et al., 2006; Walton et al., 2013). Additionally, short days impair spatial learning and hippocampal long-term potentiation, reduce hippocampal volumes, alter hippocampal dendritic complexity, and reduce hippocampal neurogenesis (Pyter et al., 2005b; Walton et al., 2011, 2013, 2014). In tandem with changes in the hippocampus, white-footed mice alter neuronal spine densities within the basolateral amygdala in response to short photoperiods, and these changes are associated with enhanced fear memory (Walton et al., 2012).

These alterations in brain function are likely preceded or mirrored by changes in brain vascularity, as the brain is an energetically expensive organ (Engl and Attwell, 2015), and any reduction in size or vascular perfusion would confer significant energetic savings. To test this hypothesis, we maintained adult male *P. leucopus* mice in long- or short-photoperiod conditions for 8 weeks, and then assessed capillary density and cerebral blood flow via fluorescein isothiocyanate (FITC)-lectin perfusion and laser speckle flowmetry (LSF). We further laser captured FITC^+^ hippocampal endothelial cells from brain tissue and assessed the expression of genes related to vascular remodeling. We predicted that animals maintained under short-day conditions would reduce cerebrovascular density and flow compared with their counterparts maintained in long day (LD) conditions, and changes in these measures would be accompanied by altered gene expression profiles in cerebral capillaries.

## Materials and Methods

### Animals

Adult (>8 weeks old), male, white-footed mice (*P. leucopus*) were purchased from the *Peromyscus* Genetic Stock Center (University of South Carolina, Columbia, SC; RRID: SCR_002769). These mice were born into long-photoperiod (16 h light/8 h dark) conditions and maintained in this lighting condition until shipment to our laboratory. Upon arrival at our facility, mice were allowed to recover from the stress of shipping and then assigned randomly to either SDs (8 h light/16 h dark) or LDs (16 h light/8 h dark). Mice were singly housed and maintained in their experimental lighting conditions for 8–9 weeks. Throughout the course of the experiment, mice were supplied with *ad libitum* filtered tap water and chow (catalog #7912, Harlan Teklad), a cotton nestlet, and a piece of plastic housing enrichment. Weekly cage changes and body mass measures were the only physical disturbances throughout the experiment. Following each experiment, the brain (cohort 1), seminal vesicles (cohort 2), and paired testes were dissected. Organ masses were taken using an analytical balance (AE 240, Mettler Toledo). All procedures and experiments described below were approved by our affiliated Institutional Animal Care and Use Committee. Cohort 1 was used for lectin, immunohistochemistry, and laser capture experiments (described below). From this cohort, we originally started with 11 mice in the LD condition and 10 mice in the SD condition. During lectin perfusion, two LD mice had cardiac arrest prior to 5 min of lectin circulation and were excluded from analyses (LD = 9). Of the remaining mice in LD and SD conditions, four were randomly selected from each group for lectin visualization alone. The remaining samples were conserved to enable laser capture microdissection (LCM) studies. For LCM, sufficient sample from two SD lectin mice remained to include with untouched sample blocks (eight SD mice for LCM, five LD mice for LCM). After LCM, three blocks from the SD and LD groups remained for matrix metalloproteinase 2 (MMP2) staining of hippocampus (three SD mice for MMP2 staining, three LD mice for MMP2 staining). Cohort 2 was used for laser speckle flowmetry (LSF), where no mice were excluded from analyses (nine SD mice, eight LD mice). All mice were used for somatic and reproductive tissue measurements. Only mice in cohort 1 had their brains weighed, and only mice in cohort 2 had their seminal vesicles weighed, as this is the most robust measure of reproductive regression in response to photoperiod.

### Blood vessel quantification

**Figure 1.**
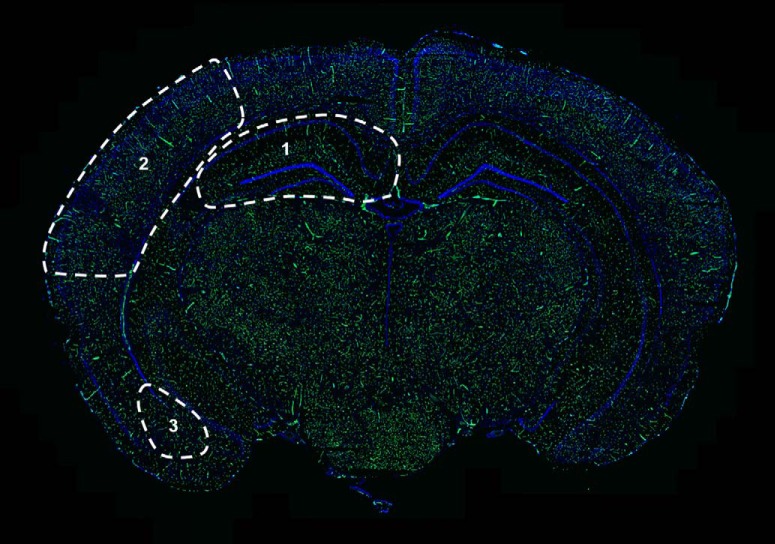
Representative 12 μm coronal section of *P. leucopus* brain. Regions used for FITC–lectin blood vessel quantification are delineated as hippocampus (1), cortex (2), and amygdala (3). The section is counterstained with DAPI.

After 8 weeks in their respective photoperiod conditions, mice were deeply anesthetized via intraperitoneal injection of 120 mg/kg ketamine and 24 mg/kg xylazine in a vehicle containing 0.9% sodium chloride. Upon sedation, mice were transcardially perfused with 250 μl of FITC-tagged tomato (*Lycopersicon esculentum*) lectin over the course of 1 min (0.5 mg/ml; Vector Laboratories). This lectin binds to complex-type *N*-glycans glycoproteins found on the luminal surface of capillary endothelial cells, enabling morphological visualization and quantification specifically of capillary bed features (Robertson et al., 2015). This technique specifically allows for the visualization of patent (open) vessels and leaves inactive vessels unstained (Inai et al., 2004). Importantly, cerebral capillary density is recognized as a key variable in the study of brain angioplasticity in response to metabolic acclimatization (Boero et al., 1999; Benderro et al., 2012; Benderro and LaManna, 2014). To that end, our lectin perfusion approach was not designed for staining/quantification of large arteries or veins, but speaks to adaptive capillary remodeling in response to changes in photoperiod. FITC–lectin was allowed to circulate for 5 min, after which the mouse was decapitated; and brains were dissected, weighed, embedded in O.C.T. compound (Sakura), and flash frozen in liquid nitrogen. Twelve micrometer serial sections were cut on a cryostat directly onto Superfrost Plus slides. DAPI was used as a nuclear counterstain. The FITC–lectin signal in the hippocampus, cortex, and amygdala (Fig. 1, representative image) was determined by a condition-blinded observer using the AutoMeasure plug-in within Axiovert software (version 4.8, Zeiss), as described previously (Khanna et al., 2013, 2015).

### Immunohistochemistry

Immunohistochemical determination of MMP2 expression was performed as previously described (Rink et al., 2011; Khanna et al., 2013). Twelve micrometer sections were blocked in 10% normal goat serum, followed by an overnight (4°C) incubation with primary antibody (0.5 µg/ml; anti-MMP2, Abcam; RRID:AB_10864041). Signal was visualized by reaction with fluorescent secondary antibody (30 min incubation at room temperature; goat anti-rabbit Alexa Fluor 568, Life Technologies). Sections were placed in DAPI for 5 min prior to being coverslipped. Images were captured using an Axiovert 200M microscope (Zeiss), and expression was quantified as the percentage area in hippocampus, amygdala, and S1 cortex regions of interest (ROIs) using the AutoMeasure plug-in within Axiovert software (version 4.8, Zeiss; Fig. 1). ROIs were standardized across sections and localized using a mouse stereotaxic atlas (Paxinos and Franklin, 2004). Observation of no staining following omission of the primary antibody from the primary incubation acted as a negative control.

### Laser capture and quantitative PCR

The 12-µm-thick sections for LCM were mounted onto RNase inhibitor-treated thermoplastic (polyethylene napthalate)-covered glass slides (PALM). FITC–lectin perfused blood vessels from the hippocampus were collected using MicroLaser, MicroBeam, and RoboStage/RoboMover systems (PALM). More than 100,000 µm^2^ of capture elements were collected from each sample for downstream RNA isolation, cDNA synthesis, and quantitative PCR (qPCR). After laser cutting, the isolated vessels were catapulted directly into 35 µl of RNA extraction buffer (PicoPure RNA Isolation Kit, Life Technologies) situated directly above the section in a microtube cap. An additional 15 µl of extraction buffer was added after collection, and RNA was isolated from captured and catapulted elements. cDNA was synthesized from >250 ng of RNA using oligo-dT primer and Superscript III. cDNA was then quantified using SYBR Green-I in a real-time PCR reaction. Relative gene expression was standardized to 18S ribosomal RNA expression. Primer sequences are available in Table 1. These genes were chosen because they are associated with vascular remodeling, and we have previously examined many of them in the context of reproductive regression in *P. leucopus* (Pyter et al., 2005a). Verification of endothelial cell enrichment in laser-captured samples was confirmed via high expression of Von Willebrand factor (VWF), and low expression of glial fibrillary acidic protein (GFAP) and neurofilament heavy (NFH).

**Table 1:** Primer sequences used in qPCR

**Gene**	**Forward primer 5'-3'**	**Reverse primer 5'-3'**	**Tm**
*Vegf*	CCA GGC TGC ACC CAC GAC AG	TGA GGT GTG GGG GCT GCT GT	F: 63.5R: 64.1
*Hif1α*	CTG TGA TGA AAG AAT TAC TGA GTT GAT G	CAT AAA TTG AGC GGC CCA AA	F: 54.3R: 53.9
*Tgfβr3*	CAG GAC CAG CTC GAT GGA A	CAC CAG GAA GAG GTC TGT TGT TAT ACA	F: 56.9R: 58.0
*Timp1*	CAG TCC CTG CCG CCA TCG TC	TGT GGG TGG AGT GGG GCA CA	F: 63.2R: 64.1
*Clic1*	TGG ACC GAG CGG AGG GTC TG	CCA TGG TTG CGT CGG GGA CC	F: 63.8R: 63.6
*Mmp2*	CAC AAG TGG CCT GGG GAG CG	GCG TGG CTT CCG CAT GGT CT	F: 63.6R: 63.2
*Csf2*	CTG CTC CCA CTC GCT CAC CC	AGG TTG CCC CGT AGG CCC TT	F: 62.8R: 64.0
*18s*	GTA ACC CGT TGA ACC CCA TT	CCA TCC AAT CGG TAG TAG CG	F: 55.3R: 55.1
*Vwf*	CCG GAA GCG ACC CTC AGA	CGG TCA ATT TTG CCA AAG ATC T	F: 59.5R: 54.0
*Gfap*	CAC GTG GAG ATG GAT GTG GC	CAG TTG GCG GCG ATA GTC ATT A	F: 58.4R: 57.2
*Nfh*	CGA GCT GTA CGA GCG CGA GG	AGC TCG CCC ACC TCC TCC TG	F: 62.8R: 64.1

F, Forward; R, reverse.

### Laser speckle flowmetry

A second subset of mice was housed in photoperiodic (8 h light/16 h dark and 16 h light/8 h dark) and housing conditions similar to those used in the first experiment. These mice were supplied tap water and food (catalog #TD 01432-I, Harlan-Teklad, supplemented with fenbendazole) *ad libitum*. After 8–9 weeks in their respective photoperiod conditions, mice were deeply anesthetized in isoflurane (4–5% induction, 1.5% maintenance) and positioned for laser speckle flowmetry. The skin of the scalp was cut along the sagittal plane to expose the braincase for imaging. A bolus of saline was applied to the skullcap to normalize the effect of light refraction between animals. LSF recordings of the neocortex were acquired from a 1 × 1 cm field of view using a 785 nm, 80 mW laser with a sampling rate of 60 Hz at a working distance of 10 cm (PeriCam PSI HR System, PeriMed). Relative perfusion units were averaged over a 10 s sampling period.

### Statistics

Group means were compared using two-tailed independent-samples *t* tests. If groups displayed unequal variances or data was non-normally distributed, nonparametric tests (i.e., Mann–Whitney *U* test) were used. Statistical significance was set at *p* ≤ 0.05. Statistics were completed with SPSS version 22 (IBM) and visualized using Prism version 5.0 (GraphPad Software). Table 2 contains more information regarding the statistical results presented.

**Table 2:** Statistical table

Figure	Panel	*Data structure	Test type	Observed power†/*p* value
2	*A*	Normal	*t* test	0.108/*p* = 0.476
2	*B*	Normal	*t* test	0.052/*p* = 0.901
2	*C*	Normal	*t* test	0.097/*p* = 0.512
2	*D*	Normal	*t* test	0.238/*p* = 0.213
3	*D* (hippocampus)	Normal	*t* test	0.742/*p* = 0.02
3	*D* (cortex)	Non-normal	Mann–Whitney *U*	0.517/*p* = 0.029
3	*D* (amygdala)	Normal	*t* test	0.052/*p* = 0.871
4	*B*	Normal	*t* test	0.619/*p* = 0.027
5	*A* (MMP2)	Non-normal	Mann–Whitney *U*	0.635/*p* = 0.019
5	*B*	Normal	*t* test	1/*p* = 0.00019

*Normality tested using Shapiro-Wilk Test.

†Observed power calculated with G*Power version 3.1.7; statistical analyses were completed with SPSS Statistics (IBM) version 22.

## Results

### Somatic and reproductive masses

**Figure 2.**
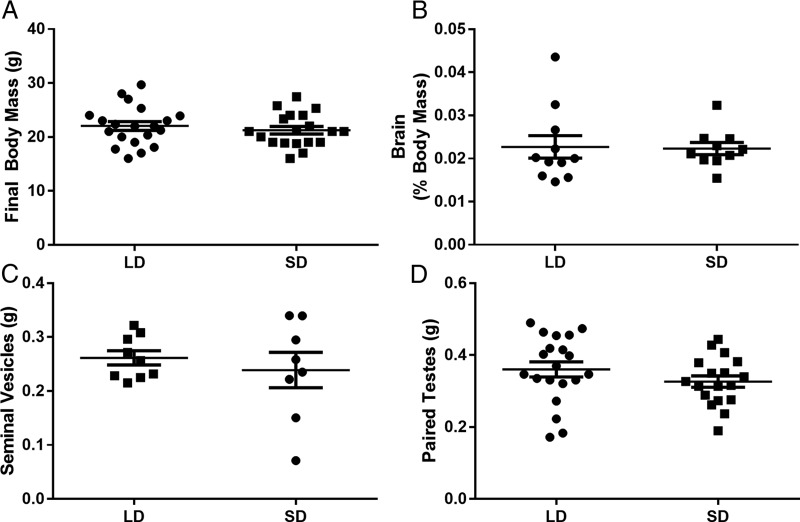
***A–D***, Eight weeks of short-day exposure did not cause gross body mass loss (***A***), reduction in brain mass (***B***), or reproductive regression (***C***, ***D***), as measured by seminal vesicle and testes masses (note: brain weights were measured only for cohort 1, and seminal vesicle weights were measured only for cohort 2). Error bars represent the SEM.

After 8 weeks in their respective photoperiod conditions, both cohorts of mice did not differ in body mass (cohort 1: *t =* 1.596, *p* = 0.126; cohort 2: *t* = 0.6, *p* = 0.557), brain mass (cohort 1: *t* = 0.913, *p* = 0.373), brain mass corrected for body mass (cohort 1: *t* = 0.126, *p* = 0.901), testes mass (cohort 1: *t* = 0.649, *p* = 0.524; cohort 2: *t* = 1.196, *p* = 0.250), or mass of the seminal vesicles (cohort 2: *t* = 0.672, *p* = 0.512; Fig. 2). These data indicate that reproductive responses to photoperiod were not apparent, suggesting a photoperiod-nonresponsive phenotype (Majoy and Heideman, 2000). Alternatively, previous reports have suggested that an exposure of 10–12 weeks elicits a maximal photoperiodic response (Pyter et al., 2005a; Walton et al., 2012).

### Short photoperiods reduce central capillary density and cerebral blood flow

**Figure 3.**
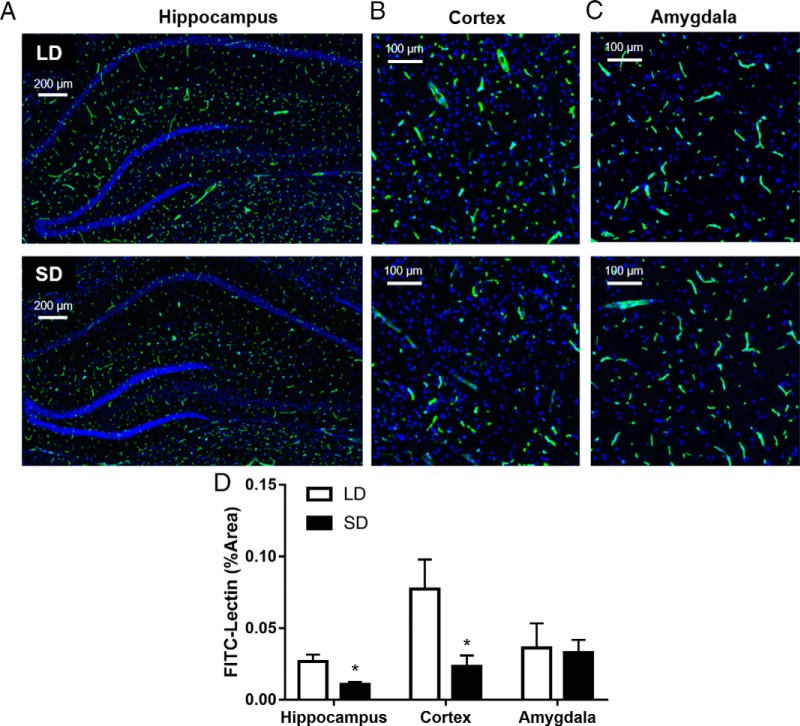
Short days reduce blood vessel density in the hippocampus and cortex. ***A–C***, Representative images of hippocampus (***A***), cortex (***B***), and amygdala (***C***) in LD (top row) and SD (bottom row) mice. Quantification of the mean ± SEM per group are presented for these brain regions in ***D***. *p < 0.05. *N* = 4/group.

**Figure 4.**
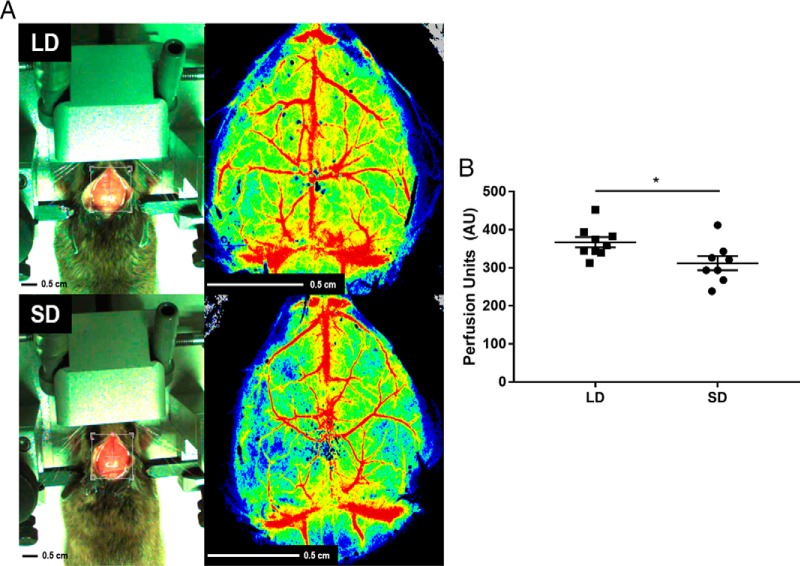
Short days reduce cerebral blood flow by ∼15%. ***A***, Representative speckle contrast images of the brain of an LD and an SD mouse. ***B***, Quantification of relative perfusion units between LD and SD animals. *N* = 9 LD, 8 SD. Error bars represent the SEM. *p < 0.05.

Mice maintained in short-day conditions had reduced FITC–lectin signal in the hippocampus (SD mean, 0.0108 ± 0.003; LD mean, 0.0268 ± 0.009; *t* = 3.13, *p* = 0.02) and cortex (SD mean, 0.0232 ± 0.015; LD mean, 0.0775 ± 0.041; *U =* 16, *p* = 0.029), but not the amygdala (SD mean, 0.033 ± 0.0175; LD mean, 0.0363 ± 0.034; *t* = 0.169, *p* = 0.871; Fig. 3) compared with their LD counterparts. Furthermore, SD mice had an ∼15% reduction in cortical blood flow compared with their LD counterparts (SD mean, 311.95 ± 52.44; LD mean, 367 ± 40.37; *t* = 2.442, *p* = 0.027; Fig. 4). These data provide evidence that short photoperiods decrease hippocampal and cortex capillary density, suggesting reduced cortical blood flow.

### Short photoperiods increase hippocampal MMP2 expression

**Figure 5.**
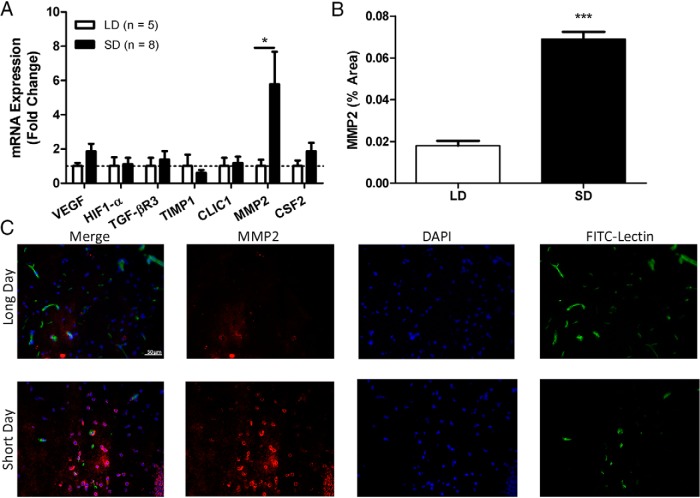
Short days increased MMP2 mRNA expression in brain endothelial cells and MMP2 protein in the hippocampus. ***A***, Gene expression in laser-captured hippocampal FITC^+^ endothelial cells reveal higher MMP2 expression in short days. ***B***, Immunohistochemical quantification of MMP2 staining in hippocampus (*N* = 3/group). ***C***, Representative immunofluorescent images of an LD and an SD housed mouse stained with anti-MMP2 primary Ab, DAPI, and FITC-lectin. Error bars represent the SEM. *p < 0.05, ***p < 0.001.

Hippocampal FITC^+^ blood vessels from SD mice expressed higher levels of the gelatinase MMP2 (fold change from LD, 5.77 ± 5.37; *U* = 4, *p* = 0.019) compared with LD mice (Fig. 5*C*). No changes were detected in any of the other gene candidates examined (*p* > 0.05 in all cases). Additionally, SD mice increased MMP2 expression in the hippocampus (Fig. 5*B*) compared with LD mice (SD mean, 0.069 ± 0.0063; LD mean, 0.02 ± 0.00085; *t* = 13.18, *p* = 0.00019). We focused on the hippocampus for these measures because a large amount of research has demonstrated short-day reductions in hippocampal function and hippocampal-dependent tasks (Pyter et al., 2005b; Walton et al., 2013). These data indicate that short-day exposed mice had greater hippocampal expression of MMP2 at the mRNA and protein level compared with LD mice.

## Discussion

Together, these data demonstrate that day length can alter brain blood vessel dynamics in adult mammals. Mice maintained in short-day conditions had decreased cortical and hippocampal perfusion (Figs. 3, 4) and increased MMP2 mRNA expression in brain capillaries and MMP2 in the brain parenchyma (Fig. 5).

Previous research has examined other environmental contributors that increase central blood vessel formation. It is well documented that environmental enrichment or motor activity can increase central angiogenesis and synaptogenesis in rats (Black et al., 1987, 1990; Sirevaag et al., 1988; Ding et al., 2006). Environmental contributors that decrease central blood flow, however, remain undefined. *P. leucopus* display seasonal changes in cognitive capacity; short days decrease performance on hippocampal dependent tasks, hippocampal neurogenesis, and dendritic spine densities (Pyter et al., 2005b; Walton et al., 2014). Our data suggest that this phenomenon is accompanied by capillary remodeling in the hippocampus and cortex; thus, short-day reductions in blood flow may precede declines in brain function. It is important to note that due to limitations in the penetration depth of the laser speckle instrument, we were unable to reliably measure hippocampal-specific blood flow using this method. However, changes in blood flow within the hippocampus are evident as FITC-lectin binds only active capillary beds when administered via transcardial perfusion, leaving inactive blood vessels unstained. Additionally, because our lectin perfusion method selectively stains capillary beds (Robertson et al., 2015), it was important to test whether changes in these microvessels resulted in measurable changes in blood flow.

Regulation of testicular angiogenesis in *P. leucopus* has been studied in the context of photoperiod-induced changes in reproduction. In the testes, short days trigger the expression of *Hif1α*, *Serpine1*, and *Tgfβr3*, inhibiting angiogenesis and halting reproductive function during testicular regression (Pyter et al., 2005a). Reduced blood flow to the brain coincident with gonadal regression may allow for animals to trade off reproductive function and cognitive capacity for somatic maintenance and thermogenesis during the impending winter months. We found no evidence of reproductive regression in the present study, indicating that changes within the brain can occur independently from photoperiodic regulation of the reproductive system.

MMPs act to degrade a variety of extracellular matrix proteins and participate in vessel remodeling, apoptosis, cell migration, proliferation, and host defense (Chakraborti et al., 2003). MMP2 (gelatinase A) primarily catalyzes the breakdown of type IV collagen, a component of endothelial basement membranes. In the brain, MMP2 can be produced by neurons, glia, and endothelial cells (Planas et al., 2001). It also plays a large role in endometrial remodeling during menses and the regulation of vascularization in response to immune activation (Freitas et al., 1999; Parks et al., 2004). MMP2 is specifically implicated in seasonal changes in reproductive function in Siberian hamsters (*Phodopus sungorus*), another seasonally breeding small rodent (Salverson et al., 2008; Shahed et al., 2015a,b). In this species, MMP2 facilitates photostimulated ovarian recrudescence after extended short-day exposure. That is, MMP2 is rapidly induced in the ovaries after transfer from short to long photoperiods. It is interesting to note that MMP2 in the ovaries of *P. sungorus* is elevated in response to long photoperiods, while in the brains of *P. leucopus* that were used in our study it was increased in response to short photoperiods. Although widely implicated in angiogenesis, the initial steps of MMP2 action involve the fragmentation of the capillary basal lamina, leading to downstream migration of endothelial cells in response to angiogenic factors (Sang, 1998). In *P. leucopus*, the initial steps of basement membrane breakdown occur (as evidenced by increased MMP2 expression), but the subsequent formation of functional blood vessels seems to be impaired in SD mice (reduced FITC–lectin signal). In support of this, we found no evidence of increased vascular endothelial growth factor (VEGF) expression in short-day mouse brain endothelium (Fig. 5*A*).

In sum, our data demonstrate a role of photoperiod in the regulation of central blood flow in an adult mammal. Further experiments should address whether the transition back into a long photoperiod reverses short day-induced vessel reductions, the time course of blood vessel remodeling, and the role that melatonin or other seasonally regulated molecules play in the phenotype we observed. Additionally, these data provide an impetus for investigating seasonal rhythms in cardiovascular disease (Ricci et al., 1992; Sheth et al., 1999), because the short days of winter may be an overlooked but important contributor, although this remains to be determined. It is important to note that our data represent a “snapshot” of the dynamic changes occurring in response to short photoperiods; and because we did not observe reproductive regression, our findings may represent an intermediate phenotype. However, independent photoperiodic regulation of the reproductive system and the brain has been described in a closely related species of California mice (*Peromyscus californicus*), where short photoperiods induce aggressive behavior without reproductive regression, a phenotype that can be recapitulated with the administration of exogenous melatonin (Nelson et al., 1995; Laredo et al., 2014). Additionally, there is high heritability in reproductive nonresponsiveness to short photoperiods in *P. leucopus* (Heideman et al., 1999), and photoperiodic responsiveness within the same *Peromyscus* species depends on their latitude of origin (Dark et al., 1983). Furthermore, short photoperiod-induced changes in hippocampal neurogenesis are evident in as few as 2 weeks of short-photoperiod exposure, well before reproductive regression occurs (Walton et al., 2014). These data indicate that changes within the CNS of *Peromyscus* species can be uncoupled from alterations in the reproductive system.

## Synthesis

The decision was a result of the Reviewing Editor Rae Silver and the peer reviewers coming together and discussing their recommendations until a consensus was reached. A fact-based synthesis statement explaining their decision and outlining what is needed to prepare a revision is listed below. The following reviewer(s) agreed to reveal their identity: Michael Lehman

The rebuttal letter is very clear and well documented with suitable references. However, some of the points made in the rebuttal letter are not included in the revised manuscript. It is necessary to provide the missing information in order to strengthen the manuscript.

The following information appears to be lacking in the revised manuscript.

1)A more detailed quantification (per vessel type: arteries, veins, cortical penetrating vessels, capillaries) seems fundamental to understand the vascular remodeling dynamics. This might require additional time-points along the 8 weeks (and in the reversal process, as in comment above).

FITC-tagged tomato (Lycopersicon esculentum) binds to complex-type N-glycans glycoproteins found on the luminal surface of capillary endothelial cells, enabling morphological visualization and quantification of capillary bed features (Robertson et al, 2015). ....etc

2)It is unclear why some animals were excluded from analysis. The rebuttal makes the numbers of animals clear, and this information should be provided somewhere in the text. It is not clearly explained at present.

We apologize for the lack of clarity in this regard, and have amended the manuscript to include reasoning for the number of animals used in each analysis. Cohort 1 was used for lectin, MMP2, and laser capture experiments. From this cohort, we originally started with 11 mice in the LD condition, and 10 mice in the SD condition. During lectin perfusion, 2 LD mice had cardiac arrest prior to 5min of lectin circulation and were excluded from analyses (LD = 9). Of the remaining mice in LD and SD conditions, 4 were randomly selected from each group for lectin visualization alone. Remaining samples were conserved to enable laser capture microdissection (LCM) studies. For LCM, sufficient sample from 2 SD lectin mice remained to include with untouched sample blocks (SD = 8, LD = 5 for LCM). After LCM, 3 blocks from SD and LD groups remained for MMP2 staining of hippocampus (SD = 3, LD = 3 for MMP2 staining). Cohort 2 was used for laser speckle flowmetry, where no mice were excluded from analyses (SD = 9, LD = 8). All mice were used for somatic and reproductive tissue measurements. Only mice in cohort 1 had their brains weighed, and only mice in cohort 2 had their seminal vesicles weighed, as this is the most robust measure of reproductive regression in response to photoperiod.

3)Please explain what an "active" blood vessel might be in contrast to an "inactive" blood vessel, and provide a reference.

We apologize for the lack of clarity on this subject. Because the FITC-tagged tomato lectin only travels through (and binds) active capillary vasculature when transcardially perfused, it is a reliable index of blood flow (i.e., it does not reach inactive vessels).

4)Some references in the rebuttal letter are omitted from the revised text. Please correct or explain the rationale for the omissions.( For example, Heideman et al 1999).
